# Upregulation of KLHDC4 Predicts a Poor Prognosis in Human Nasopharyngeal Carcinoma

**DOI:** 10.1371/journal.pone.0152820

**Published:** 2016-03-31

**Authors:** Yi-Fan Lian, Jing Yuan, Qian Cui, Qi-Sheng Feng, Miao Xu, Jin-Xin Bei, Yi-Xin Zeng, Lin Feng

**Affiliations:** 1 Department of Experimental Research, Sun Yat-sen University Cancer Center, State Key Laboratory Oncology in South China, Collaborative Innovation Center of Cancer Medicine, Guangzhou, China; 2 Beijing Hospital, Beijing, China; The University of Hong Kong, HONG KONG

## Abstract

Kelch proteins are implicated in the pathogenesis of many human diseases, including cancer. Nasopharyngeal carcinoma (NPC) is a rare malignancy in most countries, but prevalent in southern China and certain areas of Southeast Asia. In this study, we identified Kelch Domain Containing 4 (KLHDC4), an orphan member of the kelch repeat superfamily, as a prognosis marker for NPC. We examined the expression of KLHDC4 in 168 NPC cases by immunohistochemical staining and found a substantially higher level of KLHDC4 in NPC biopsies compared to adjacent normal nasopharyngeal mucosa. KLHDC4 expression was significantly related to the T classification (*P <*0.05), N classification (*P <*0.05) and total staging (*P <*0.01) in NPC, and patients with higher KLHDC4 expression had poorer overall (*P <*0.01) and metastasis-free survival (*P <*0.05) rates. Knockout (KO) of KLHDC4 via CRISPR/Cas9-mediated gene editing in NPC cell line dramatically inhibited cell proliferation, colony formation in soft agar and tumor formation in nude mice. In addition, cell migration and invasion were also impaired by KLHDC4 depletion as revealed by wound healing and Transwell assay. Mechanically, loss of KLHDC4 markedly induced spontaneous apoptosis in NPC cells, as evidenced by increased levels of cleaved caspase-3 and cleaved PARP. Consistently, KLHDC4 knockout cell-derived xenografts also showed elevated cleaved caspase-3 and PARP but reduced Ki-67 staining. In conclusion, our results suggest that KLHDC4 promotes NPC oncogenesis by suppressing cellular apoptosis. Thus, KLHDC4 may serve as a prognosis biomarker and a potential therapeutic target for NPC.

## Introduction

Nasopharyngeal carcinoma, a malignant tumor arising from the epithelium of the nasopharynx [1], shows a remarkable racial and geographical distribution: it is rare in most parts of the world but occurs with high frequency in Southeast Asia, particularly in southern China, with an incidence of more than 20 cases per 100,000 population per year [2]. Interplay among several factors, including genetic susceptibility, Epstein-Barr virus (EBV) infection and dietary exposure to nitrosamines and nitrites during childhood, are believed to contribute to the etiology of NPC [3, 4]. Although 5-year survival rates of 80% can be achieved with the typical treatment of high-dose radiotherapy plus adjunctive chemotherapy, recurrence and metastasis still occur and are the main causes of treatment failure [5, 6]. Therefore, identifying molecular biomarkers for NPC prognosis and progression and developing an innovative targeted therapy are urgently needed.

Apoptosis, which is essential for maintaining tissue homeostasis by eliminating unnecessary or deleterious cells, is a cell suicide program that is under precise, multi-stage regulation. There are essentially two distinct pathways for triggering apoptosis: the death receptor pathway mediated by procaspase-8 and the mitochondrial pathway involving procaspase-9 [7]. Through diverse stimuli, activated caspase-8, -9 can further initiate the function of downstream effectors (principally caspase-3, -6, -7 and PARP), which in turn cleave a variety of substrates to give rise to internucleosomal DNA fragmentation and characteristic morphological changes [8]. Suppression of apoptosis is a recognized hallmark of cancer, and because the apoptotic program can be manipulated to produce massive cell death, the genes controlling apoptosis are ideal drug targets for cancer treatment [9, 10]. Indeed, many proapoptotic compounds and proteins have been reported to be effective in killing NPC [11–15], suggesting that targeting apoptosis pathways is a promising strategy for NPC treatment.

The kelch superfamily is one of the largest evolutionarily conserved families, and the ~100 known family members have been implicated in diverse cellular functions, including cell migration, cytoskeletal arrangement, protein degradation and gene expression [16, 17]. The kelch repeat domain, which is present in all kelch proteins, is composed of five to seven tandem repeats of the kelch motif, forming a β-propeller structure that functions as a scaffold for protein-protein interactions. Kelch protein dysregulation has been reported to be associated with multiple human diseases, including cancer, skeletal muscle development and neurological disorders [18]. Moreover, recent reports have demonstrated that the kelch repeat proteins associate with the complex-type ubiquitin E3 ligases called the Cullin-RING ubiquitin ligases (CRLs) and play a role in substrate recognition of this complex [19]. However, the physiological roles and underlying molecular mechanisms of most kelch repeat proteins remain elusive.

Here, we provide evidence that KLHDC4, an uncharacterized kelch repeat containing protein, is substantially overexpressed in NPC. High level of KLHDC4 associates with poor prognosis in NPC patients. Moreover, CRISPR/Cas9-mediated knockout of KLHDC4 in NPC cells results in a decrease in tumor cell growth and migration and is accompanied with increased apoptosis both *in vitro* and *in vivo*.

## Materials and Methods

### Reagents

The reagents used were as follows: mouse monoclonal antibodies against Flag (F3165, Sigma-Aldrich), GAPDH (KC-5G4, Kangcheng Biotech); rabbit monoclonal antibody against HA (3724, Cell Signaling Technology); rabbit polyclonal antibodies against KLHDC4 (HPA041665, Sigma-Aldrich), cleaved caspase-3 (9664, Cell Signaling Technology), cleaved PARP (5625, Cell Signaling Technology), Ki-67 (ab15580, Abcam). Horseradish peroxidase-conjugated (HRP) goat anti-mouse/rabbit secondary antibodies were purchased from Promega. Noble agar (214220) was purchased from BD. All other chemical reagents were obtained from Sigma-Aldrich, unless otherwise indicated.

### Cell cultures

The NPC cell line CNE2 was kindly provided by Dr. Chao-Nan Qian, Sun Yat-sen University Cancer Center (SYSUCC), China. Two normal nasopharyngeal epithelial cell lines NPEC-N2-Bmi1, NPEC-N5-Tert and two NPC cell lines SUNE1, SUNE2 were kindly provided by Dr. Mu-Sheng Zeng, Sun Yat-sen University Cancer Center, China. The other cell lines, NP69, CNE1, HK1, HNE1, HONE1 were obtained from SYSUCC. All cell lines were thawed from early passage stocks and passaged for less than 6 months. The normal nasopharyngeal epithelial cell lines, NP69, NPEC-N2-Bmi1 and NPEC-N5-Tert, which were immortalized by SV40, Bmi-1 and Tert, respectively, were grown in keratinocyte/serum-free medium (Invitrogen). Human NPC cell lines (CNE1, CNE2, SUNE1, SUNE2, HK1, HNE1, HONE1) were cultured in Dulbecco’s modified Eagle’s medium (DMEM, Life Technologies) supplemented with 10% fetal bovine serum (FBS, Biological Industries). Cells were grown in a humidified 5% CO_2_ incubator at 37°C and passaged using standard cell culture techniques.

### DNA mismatch-specific endonuclease (T7E1) assay

293T cells in 6-well dishes were cultured to 50–60% confluence as mentioned above. The cells were transfected with 1 μg of KLHDC4 single-guide RNA (sgRNA) plasmid (ligated into the pX330 vector; sgRNA#1: 5’-GGCGGACAGCTGTGGGTCTT-3’; sgRNA#2: 5’-CACAGCTGTCCGCCACCTTG-3’; both sgRNAs targeting the exon 5 of *KLHDC4* gene were selected from a published database [20] of predicted high-specificity protospacer adjacent motif target sites in the human exome) and 5 μl of Lipofectamine 2000 per well. As a control, cells were also tranfected with the pX330 empty vector. The cells were harvested at 72 hours post-transfection, and genomic DNA was extracted (Qiagen DNeasy Blood & Tissue Kit). A region of exon 5 of the *KLHDC4* gene was amplified with genomic DNA-specific primers (forward: 5’-TGACTGAGGACGTGCTTTCC-3’; reverse: 5’-CCACAGGAGAAGAGCTGCAA-3’). The homoduplex PCR products were denatured and rehybridized using stepdown annealing conditions to generate homo- and heteroduplexes. The mixture of duplexes was treated with T7E1 endonuclease for 20 minutes at 37°C (New England Biolabs); the reaction was stopped using 1.5 μl of 0.25 M EDTA, and the products were analyzed on a 3% agarose gel.

### Establishment of a KLHDC4 knockout cell line

CNE2 cells were cultured in 6-well dishes to 70–80% confluence and then cotransfected with 1 μg of KLHDC4 sgRNA#2 plasmid plus 1 μg of pSpCas9(BB)-2A-GFP plasmid and 5 μl of Lipofectamine 2000 per well. GFP was used as a fluorescent marker to sort the transfected cells. At 48 hours post-transfection, the cells were sorted into 96-well plates using fluorescence-activated cell sorting (FACS) with a Beckman-Coulter MoFlo XDP device. Single cells were validated as KLHDC4 knocked-out clone by western blotting and Sanger sequencing and then expanded as the KO cell line.

### Cell proliferation assay

Cells were seeded in 96-well plates in DMEM medium containing 10% FBS at a density of 2000 cells per well and incubated for 1–4 days. At each time point, a 10-μl aliquot of the CCK-8 solution (Dojindo) was then added to each well, and the cells were cultured for another 2 hours. The absorbance (OD value) at 450 nm was then measured using a spectrometer (SpectraMax M5 Microplate Reader, Molecular Devices LLC).

### Soft agar colony formation assay

A 2-ml layer of 0.5% agar (wt/vol) in DMEM with 5% FBS was poured into 6-well plates. Cells were resuspended in 0.35% agar (wt/vol) in DMEM with 10% FBS at a density of 10,000 cells/ml, and 1 ml of the cell suspension was poured on top of the base layer; the suspension was allowed to solidify and incubated at 37°C in 5% CO_2_ for 14 days. The number of colonies were monitored manually using an Olympus IX71 microscope.

### Wound healing assay

Cells were seeded in 6-well plates and grown to confluence. Wounds were created by scrapping the monolayer cells with a 10-μl pipette tip, and non-adherent cells were washed off using medium. The cells were observed and photographed via microscopy after 20 hours. The wound distances were measured, and the results are expressed as the average percent of wound closure compared with time zero. Image-Pro Plus 6.0 software was used to quantify the wound area.

### Transwell invasion assay

Cells were trypsinized and pelleted by centrifugation. After washing twice in phosphate buffered saline (PBS buffer), the cells were resuspended in serum-free DMEM medium at a density of 4 × 10^5^ cells/ml, and 200 μl of the cell suspension was seeded onto the basement Matrigel-coated membrane matrix (BD Biosiences). Fetal bovine serum was added to the lower chamber as a chemoattractant. After 20 hours, the noninvading cells were gently removed with a cotton swab. Invasive cells located on the lower side of the chamber were fixed with 4% paraformaldehyde (PFA) for 20 minutes at room temperature prior to Crystal Violet (C01201, Beyotime) staining. Three independent visual fields were examined via microscopic observation, and the number of cells was determined.

### Morphological analysis of apoptotic cells

The study was carried out in 6-well plates with a glass coverslide. Cells were fixed with 4% PFA and permeabilized with 0.1% Triton X-100. Then, the cells were incubated for 5 min with 4’, 6-diamidino-2-phenylindole (DAPI, BD5010, Bioworld) according to the manufacturer’s protocol. The stained cells were imaged with a fluorescent microscope (Olympus IX73).

### Flow cytometry

Cells were stained with annexin V-FITC and PI (KGA108, KeyGEN) and evaluated for apoptosis by flow cytometry according to the manufacturer’s protocol. Briefly, 1 × 10^6^ cells were washed twice with PBS and stained with 5 μl annexin V-FITC and 10 μl PI in 1 × binding buffer for 15 minutes at room temperature in the dark. Apoptotic cells were determined using a Beckman-Coulter Flow Cytometry FC500. Both early (annexin V-positive/PI-negative) and late (annexin V-positive/PI-positive) apoptotic cells were included when assessing cell death.

### Western blotting

Cells were lysed in NETN buffer (20 mM Tris-HCl at pH 8.0, 100 mM NaCl, 1 mM EDTA, 0.5% Nonidet P-40) containing 50 mM β-glycerophosphate (14405, Merck), 1 μg/ml pepstatin A (P5318, Sigma-Aldrich) and 10 μM leupeptin (L2884, Sigma-Aldrich). The lysate protein concentration was measured using the BCA protein assay kit (Pierce); after normalization to equal amounts, proteins were separated by 12% SDS-PAGE, transferred to PVDF membranes and probed with the indicated primary antibodies. The blots were then incubated with species-specific HRP-conjugated secondary antibodies, and the immunoreactive bands were visualized by enhanced chemiluminescence (ECL, Pierce).

### Immunoprecipitation

Cells were washed with ice-cold PBS buffer and then lysed in NETN buffer at 4°C for 10 min. Crude lysates were cleared by centrifugation at 14,000 rpm and 4°C for 10 min, and supernatants were incubated with S-protein agarose (69704, Novagen). The immunocomplexes were washed three times with NETN buffer and then subjected to SDS-PAGE. Western blotting was done using the antibodies specified in the figures.

### *In vivo* animal studies

All mice were handled according to the Guide for the Care and Use of Laboratory Animals. The procedures were approved by the Institutional Animal Care and Use Committee of Sun Yat-sen University Cancer Center (Reference number: 00090462). Female BALB/c nude mice (Hunan Slac Jingda Laboratory Animal Co., Ltd., Hunan, China) aged 6 weeks were used for tumor xenografts. All the animals were housed in standard cages (4 animals per cage) under specific pathogen-free conditions. Rodent laboratory chow and tap water were provided ad libitum and maintained under controlled conditions with a temperature of 24 ± 1°C, humidity of 50 ± 10%, and a 12: 12h light/dark cycle. Food and water were freely available throughout the experiments. The nude mice were randomly divided into two groups (8 mice per group), CNE2 control group and KLHDC4 KO group, respectively. Cells (5 × 10^5^ in 0.2 ml PBS) were injected subcutaneously into the left dorsal flank of nude mice. Mice were monitored every 12 hours for the first 3 days after inoculation of tumor cell lines, then daily thereafter. Tumor sizes were measured every 3–4 days. Mice were killed 4 weeks post-injection via cervical dislocation, and tumors from the two groups were extracted and weighed. The perpendicular diameters of the tumors were measured using a caliper, and the tumor volume were calculated using the formula: tumor volume (V) = π/6 × large diameter × smaller diameter^2^. The following are general humane endpoints for animals that require euthanasia in this study: 20% decrease in normal body weight; the inability to reach food or water for more than 24 hours; a tumor burden greater than 10% body weight or a tumor that exceeds 20 mm in any one dimension. All efforts were made to minimize animal suffering.

### Patient selection and immunohistochemical (IHC) staining

We evaluated the prognostic relevance of KLHDC4 expression in a cohort of 168 NPC patients who had undergone definitive treatment with curative intent at our institute from 2005 to 2013. The present study was reviewed and approved by the Institutional Review Board of Sun Yat-sen University Cancer Center, and all participants provided written informed consent.

The NPC clinical samples were fixed in 10% formalin and embedded in paraffin, and we deparaffinized and rehydrated sections of the embedded specimens. For IHC staining, deparaffinized slides were retrieved with the appropriate antigen, and endogenous peroxidase activity was blocked with 1% H_2_O_2_ in PBS for 30 min. The slides were then exposed to an anti-KLHDC4 antibody at 4°C overnight. Immunostaining was performed using Envision System with diaminobenzidine (Dako). A semi-quantitative scoring criterion was used for the IHC results, whereby both the staining intensity and positive areas were recorded. A staining index (values 0–7), obtained as the intensity of KLHDC4-positive staining (negative, 0; weak, 1; moderate, 2; or strong, 3 scores) and the proportion of immunopositive cells of interest (<25%, 1; 25–50%, 2; 50–75%, 3; ≥75%, 4 scores), was calculated. All scores were subdivided according to a cutoff value of the ROC curve in the study cohort into two categories: low expression (≤4.5) and high expression (>4.5).

### Statistical analyses

The SPSS software version 19.0 and GraphPad Prism 5 software were used to perform the statistical analyses. Correlation of the KLHDC4 staining intensity to clinicopathological characteristics was measured using Pearson’s Chi-Square or Fisher’s exact test. Cumulative survival was calculated by Kaplan-Meier analysis, and comparison between groups was achieved using the log-rank test. Each experiment was performed three times in triplicate. The significance of variances between groups was determined by the t-test. All statistical tests were two-sided, and *P <*0.05 was considered statistically significant.

## Results

### KLHDC4 is overexpressed in NPC tissue and cell lines

**Fig 1:**
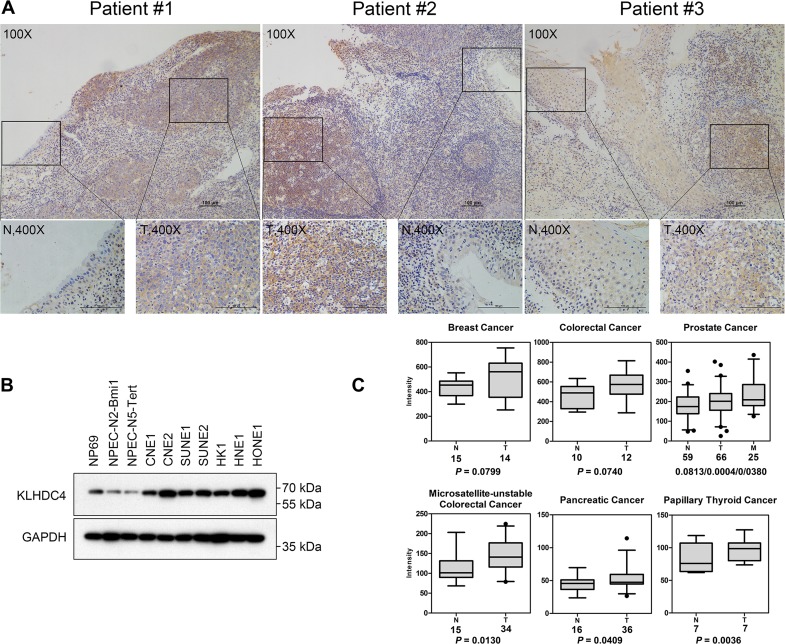
KLHDC4 is overexpressed in NPC tissue and cell lines. (A) Immunohistochemical image of three NPC samples containing both normal adjacent epithelial and tumor tissues stained with an anti-KLHDC4 antibody. N: normal adjacent epithelial tissues; T: tumor tissues. (B) Western blot of KLHDC4 expression in the above cell lines with GAPDH as the loading control. (C) Increased levels of KLHDC4 mRNA are commonly detected in various types of human cancers, as revealed by analyzing Gene Expression Omnibus microarray datasets. The line inside the boxes represents the median value. The box length indicates the interquartile range. The solid round dots have values > 1.5 interquartile ranges but < 3 interquartile ranges from the end of the box. N: normal tissues; T: tumor tissues; M: metastatic tissues. The numbers under the tissue type indicate the total cases of each cancer type. The *P* values separated with slashes indicate comparisons of normal/tumor, tumor/metastatic and normal/metastatic tissues.

To reveal a potential role for KLHDC4 in NPC, we performed immunohistochemical (IHC) analysis of KLHDC4 expression in patient-derived NPC biopsies. The KLHDC4 protein was detected in the cytoplasm of both normal nasopharyngeal epithelia and carcinoma cells. Among all primary tumor samples examined, 84 samples had both tumor and normal epithelial tissues on the same slides. The KLHDC4 expression levels in both tumors and adjacent normal nasopharyngeal tissues were scored and positive index was calculated. The positive rate of KLHDC4 in tumor tissues is 68.9% (51/84), and that in corresponding adjoining normal nasopharyngeal epithelia is 40.5% (30/84; χ^2^ = 12.027, *P <*0.001; Table 1). The intensity of KLHDC4 staining was significantly higher in NPC than in the adjacent non-malignant nasopharyngeal epithelia (Fig 1A). We also examined KLHDC4 expression in 10 nasopharyngeal epithelial cell lines, including 3 immortalized normal nasopharyngeal epithelial lines (NP69, NPEC-N2-Bmi1 and NPEC-N5-Tert) and 7 nasopharyngeal cancer lines (CNE1, CNE2, SUNE1, SUNE2, HK1, HNE1 and HONE1). The result showed dramatic increases of KLHDC4 in all NPC cell lines compared to the three immortalized normal epithelial cell lines (Fig 1B). To further assess the prognostic significance of KLHDC4 in human cancers, we searched the NCBI Gene Expression Omnibus (GEO) repository and found elevated KLHDC4 mRNA levels in tumor samples compared to normal controls in breast (GSE#9574), colorectal (GSE#4107 and GSE#24514), prostate (GSE#6919), pancreatic (GSE#16515) and thyroid (GSE#3678) cancers (Fig 1C). These results suggested that upregulation of KLHDC4 expression occurs only not in NPC but also in different types of human cancer, indicating that KLHDC4 may play a role in cancer development.

**Table 1:** KLHDC4 expression correlation in NPC and adjacent nasopharyngeal epithelia.

Groups	KLHDC4 High	KLHDC4 Low	χ^2^	*P*
Tumor	51 (68.9%)	23 (31.1%)		
Adjacent epithelia	30 (40.5%)	44 (59.5%)	12.027	0.001

### Elevated KLHDC4 expression correlates with poor prognosis in NPC patients

**Fig 2:**
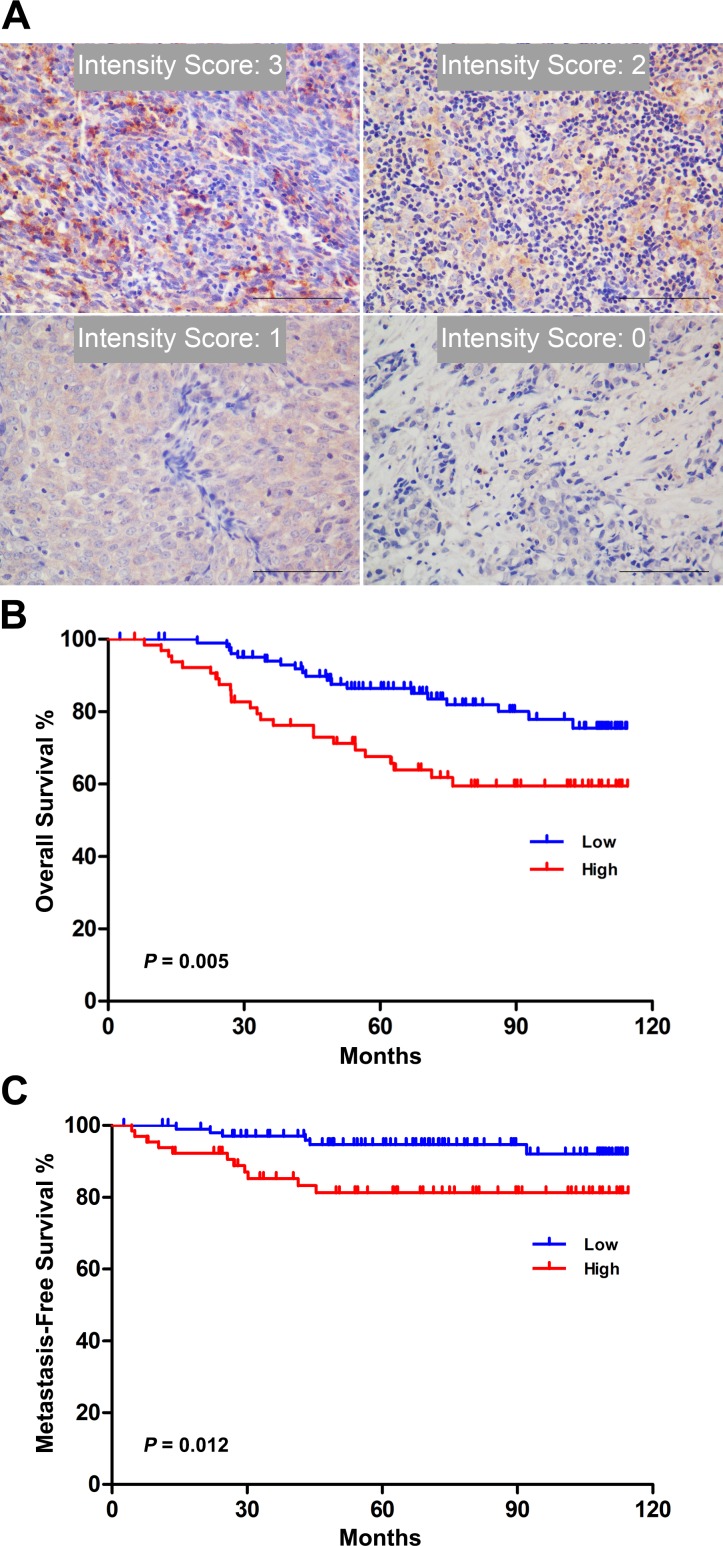
Elevated KLHDC4 expression correlates with poor prognosis in NPC patients. (A) Criteria for scoring of KLHDC4 expression intensity. Representative images are shown. All images were obtained and processed under identical conditions. Scale bars: 100 μm. (B-C) Kaplan-Meier analysis of KLHDC4 expression and overall survival (B) and metastasis-free survival rate (C) of NPC patients.

To investigate whether KLHDC4 expression can serve as a novel prognostic marker for NPC patients, we examined the correlation of KLHDC4 expression with clinicopathological factors including age, sex and pathological stage in 168 NPC patients. The staining intensity and distribution varied among the samples (Fig 2A), and a cutoff value according to the ROC curve was used to separate patients into two groups: a low KLHDC4 group (score ≤4.5, N = 103) and a high KLHDC4 group (score >4.5, N = 65). As shown in Table 2, KLHDC4 expression was significantly correlated with T classification (*P =* 0.016), N classification (*P =* 0.043) and total staging (*P =* 0.002). No difference was noted in KLHDC4 expression when stratified by gender (*P =* 1.000), age (*P =* 0.754), or M stage (*P =* 0.300). The Kaplan-Meier survival curves showed that the patients with high KLHDC4 expression had a significantly poorer overall survival (OS) and metastasis-free survival (MFS) when compared with patients with low KLHDC4 expression, as defined by the log-rank test (*P =* 0.005, Fig 2B; *P =* 0.012, Fig 2C).

**Table 2:** Relationship of KLHDC4 expression with clinicopathological characteristics in NPC.

Groups	No.	KLHDC4 Low	KLHDC4 High	χ^2^	*P*
***Gender***					
Male	124	76	48		
Female	44	27	17	0.000	1.000
***Age***					
≤45 years	88	55	33		
>45 years	80	48	32	0.110	0.754
***T Stage***					
I-II	66	48	18		
III-IV	102	55	47	5.974	0.016*
***N Stage***					
0-I	84	60	24		
II-III	94	53	41	4.322	0.043*
***M Stage***					
0	164	102	62		
1	4	1	3	2.277	0.300
***Total Stage***					
I-II	45	31	14		
III-IV	123	72	51	10.743	0.002*

*P<0.05

### Knockout of KLHDC4 reduces NPC cell growth

**Fig 3:**
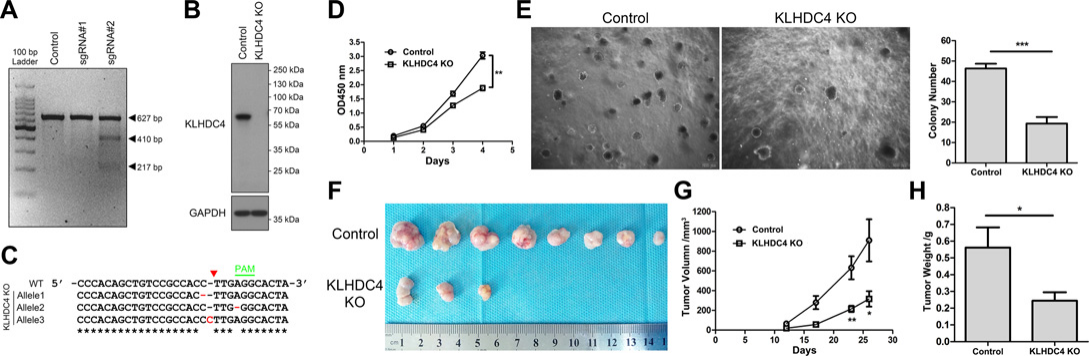
Knockout of KLHDC4 reduces NPC cell growth. (A) A T7E1 assay showed that mutation at the KLHDC4 locus was induced by transgenic Cas9 and sgRNA#2 but not sgRNA#1. (B) Western blotting confirmed that KLHDC4 was absent in KO cells. (C) Indel mutations induced by transgenic Cas9 and sgRNA#2 at the KLHDC4 locus. Representative DNA sequencing results for PCR products from KLHDC4 KO cells showing indel mutations at the targeted locus. Red dashes, deleted bases; Red bases, insertions; Asterisks, consensus bases; Red triangle, putative excision site. (D) KLHDC4 KO reduced the growth rate of CNE2 cells *in vitro*. **P <0.01. (E) KLHDC4 KO dramatically reduced colony formation in soft agar. Scale bars: 200 μm. Bars denote S.E.M. ***P <0.001. (F) All tumors isolated from mice are shown. Note none of the 8 mice inoculated with the control cells showed visible tumor growth, whereas only 3 of the 8 mice inoculated with KLHDC4 KO cells did. (G-H) Growth curves (G) and tumor weights (H) of xenograft tumors from experiments with nude mice. Changes in tumor volumes measured on the indicated days are shown. Bars denote S.E.M. *P <0.05, **P <0.01.

To gain insight into the role of KLHDC4 in the development and progression of NPC, we generated KLHDC4 knockout cells using CRISRP/Cas9-mediated gene editing technology in CNE2, a typical NPC cell line that is primarily used in NPC research. We constructed two sgRNAs targeting different regions in one of the first few exons of the human *KLHDC4* gene without obvious potential off-target effects according to bioinformatic analysis. The sgRNAs were transfected with the Cas9 expression construct into human 293T cells. Compared to empty gRNA vector, sgRNA#2 exhibited efficient cleavage activity as assessed by the T7E1 assay (Fig 3A). Therefore, sgRNA#2 was selected for generating KLHDC4 knockout cells. Western blotting showed the complete absence of the KLHDC4 protein in the KO cells (Fig 3B). We also extracted genomic DNA from these cells for PCR amplification of the sgRNA#2 target region and subcloned the PCR products into the pMD18-T vector for Sanger sequencing. Analysis of 10 independent clones revealed 3 distinct indels surrounding the protospacer adjacent motif (PAM), further validating the KO status of these cells (Fig 3C).

Next, we attempted to elucidate biological significance of KLHDC4 in NPC progression. During cell culture we noticed that KLHDC4 KO cells proliferated poorly than the wild-type counterpart. Accordingly, a significant decrease in the viability of KLHDC4 KO cells compared to control cells was observed by cell counting kit-8 (CCK-8) assay (Fig 3D). Similarly, knockout of KLHDC4 dramatically prevented colony formation by these cells in soft agar compared to control wild-type cells, indicating that anchorage-dependent growth was suppressed by KLHDC4 loss (Fig 3E). We next evaluated the *in vivo* effects of KLHDC4 deficiency on tumor growth by subcutaneous injection of parental or KLHDC4 KO CNE2 cells into nude mice and tumor growth were monitored. In contrast to the parental CNE2 cells, which formed large tumors within 4 weeks (8 of 8 mice), mice injected with KLHDC4 KO cells displayed either no tumor formation or greatly retarded tumor growth (3 of 8 mice; Fig 3F and 3G). Consistently, the average weight of the xenograft tumors were also significantly reduced in the absence of KLHDC4 (Fig 3H). These results suggest that KLHDC4 is required for NPC cell growth and proliferation both *in vitro* and *in vivo*.

### Loss of KLHDC4 reduces NPC cell migration and invasion

**Fig 4:**
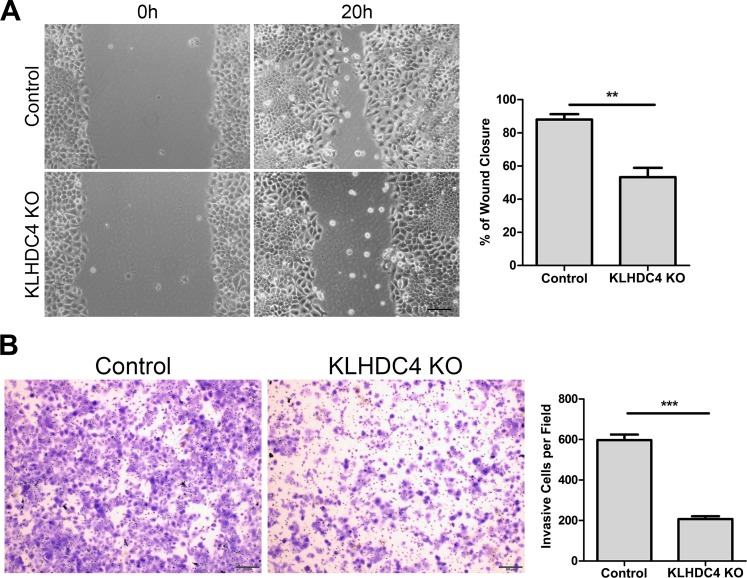
Loss of KLHDC4 reduces NPC cell migration and invasion. (A) Wound-healing assays were performed at 0 and 20 hours with control and KLHDC4 KO cells. Left panels: representative images; Scale bars: 50 μm. Right panels: quantification of the wound closure area calculated by measuring the decreaseinthe wound bed surface overtime. **P <0.01. (B) KLHDC4 KO significantly reduced the invasive ability of CNE2 cells. Left panels: representative images; Scale bars: 50 μm. Right panels: quantification of average number of cells per field. ***P <0.001.

Aberrant cell migration and invasion is frequently associated with cancer, and enhanced migration and invasion capacity is generally believed to be associated with tumor metastasis. Thus, we performed wound healing and Transwell assays to determine whether KLHDC4 expression is related to cell migration and invasion, respectively. At 20 hours after scratch injury, the KLHDC4 KO cells were able to cover only 53.3% of the scratch, whereas the control cells covered 88.0% (*P <*0.01; Fig 4A). Similarly, when allowing 20 hours for invasion, the parental CNE2 cells were able to invade through the Transwell chamber inserts, whereas invasion by the KLHDC4 KO cells was significantly attenuated (Fig 4B). Collectively, our results suggest that KLHDC4 deficiency dramatically impairs cell migration and invasion *in vitro*.

### Loss of KLHDC4 induces apoptosis in NPC cells

**Fig 5:**
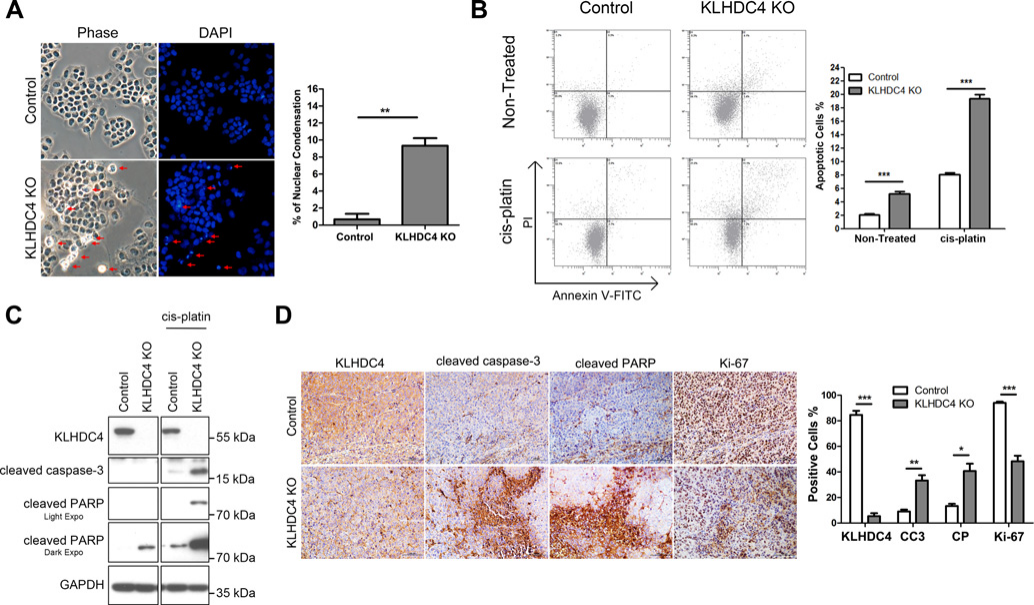
Loss of KLHDC4 induces apoptosis in NPC cells. (A) Control and KLHDC4 KO cells were stained with DAPI and photographed by fluorescence microscopy under fluorescence and light fields. Left panel: representative images; the arrows indicated the condensed or fragmented nuclear and multiblebbing cells. Right panels: quantification of cells with condensed nuclear per field. **P <0.01. (B) Representative flow cytometric analysis of apoptotic CNE2 control and KLHDC4 KO cells stained for annexin V-FITC/PI under both non-treated and cis-platin treated conditions. The results are summarized in the right panel. Bars denote the S.E.M. (C) Western blot analysis of the expression of cleaved caspase-3, cleaved PARP and GAPDH, as the loading control, under both non-treated and cis-platin treated conditions. (D) Immunohistochemical analysis of KLHDC4, cleaved caspase-3, cleaved PARP, and Ki-67 expression in xenografts generated from CNE2 control and KLHDC4 KO cells. Left panels: representative images; Scale bars: 100 μm. Right panels: quantification of average percentage of cells with positive staining per field; CC3: cleaved caspase-3; CP: cleaved PARP. ***P <0.001.

Apoptosis has long been known to be associated with tumor growth and metastasis. Induction of apoptosis is also a therapeutic strategy for the treatment of NPC. To define the underlying molecular mechanism by which KLHDC4 inhibits NPC progression, we first examined morphological and biochemical changes in cells after loss of KLHDC4. The control CNE2 cells exhibited a normal cobblestone epithelial morphology and apoptotic cells were hardly observed. In contrast, around 10% of the KLHDC4 KO cells displayed the typical characteristics of apoptosis, such as cell shrinkage, membrane blebbing, and nuclear condensation (Fig 5A). Compared to the control cells, annexin V/PI staining showed a significant increase in apoptotic cells (annexin V positive/PI negative and annexin V positive/PI positive) among the KLHDC4 KO population under both non-treated and cis-platin treated conditions (Fig 5B). In addition, the activity of caspase-3 and PARP, as demonstrated by the appearance of their cleaved forms, were markedly elevated in KLHDC4 KO cells, indicating that the cell death induced by KLHDC4 deficiency via a route of apoptosis (Fig 5C). In agreement with the aforementioned *in vitro* results, IHC of the KLHDC4 knockout xenograft biopsies showed a substantial increase in the number of cleaved caspase-3 and cleaved PARP-positive cells and decreased Ki-67 positive cells (Fig 5D), further supporting the role of KLHDC4 in promoting NPC progression through regulating apoptosis.

## Discussion

NPC is an uncommon cancer exhibiting complex interactions of multiple etiologic factors. An overall understanding of the molecular pathogenesis of this disease is still lacking, and the identification of effective prognostic and diagnostic biomarkers are urgently needed. In this study, we reported that KLHDC4, an uncharacterized kelch protein, is associated with nasopharyngeal carcinoma tumorigenesis and development.

We found that KLHDC4 is markedly upregulated in NPC clinical specimens and cell lines. Furthermore, gene expression profiles from the GEO repository also reveal upregulation of KLHDC4 mRNA in tumor sections of different origins (Fig 1). These results clearly indicated that KLHDC4 expression is involved in cancer development. However, it remains to be defined how KLHDC4 expression is regulated during pathogenesis.

Next, we focused on the potential relevance of KLHDC4 expression to clinicopathologic characteristics and its application in prognostic evaluation of NPC patients. Our data showed significant correlations of KLHDC4 expression levels with tumor staging. High KLHDC4 protein expression also predicts a poorer overall survival and metastasis-free survival in NPC patients (Fig 2). These results strongly suggest an oncogenic role of KLHDC4 in NPC development and highlight its potential use in NPC prognostic prediction.

Consistent with our observations in clinical samples, we found that knockout of KLHDC4 gene in NPC cell line reduced cell growth, migration and invasion, as well as tumorigenesis in xenograft mice model (Figs 3 and 4). Together with the clinical data, these results indicate that KLHDC4 may play an oncogenic role in NPC. CNE2 is a cell line commonly used in NPC studies due to its undifferentiated characteristic and easy maintenance [21]. However, studies of KLHDC4 gene in additional NPC cell lines are warranted to further confirm the role of KLHDC4 in NPC development.

The pathogenesis of NPC is complex and involves a number of signaling pathways related to tumor cell growth and metastasis [22]. Among them, the apoptosis signaling pathway is crucial for cells survival and tumorigenesis. Importantly, defective or inefficient apoptosis has become an hallmark of cancer cells [23]. Recently, several reports have demonstrated that dysregulation of gene expression in NPC were found to inhibit cancer cell growth and metastasis through induction apoptosis [24, 25]. Since we noticed that KLHDC4 KO cells displayed typical characteristic of apoptosis, we tried to define whether KLHDC4 participates in cellular apoptosis regulation. A higher proportion of annexin V-positive cells among the KLHDC4 KO population were found under both static and cis-platin induced conditions. Furthermore, elevated expression of cleaved caspase-3 and cleaved PARP were seen in tumor cells and xenografts after depletion of KLHDC4 (Fig 5). These data indicate that loss of KLHDC4 induces activation of cellular apoptosis in CNE2 cells and possibly hinders viability and tumor cell growth and metastasis.

We also attempted to further elucidate the downstream signal that mediates KLHDC4’s anti-apoptotic effect. Several reports highlighted that kelch proteins participate in regulating cellular apoptosis. KEAP1, a protein with BTB-BACK domain and six tandem kelch repeats, serves as a substrate adaptor of the Cullin3 E3 ligase complex. Investigations demonstrated that KEAP1 plays a key role in regulating apoptosis via modulation of the cellular redox balance by targeting NRF2, a transcription factor that is often overexpressed in many human cancers, for proteasome-mediated degradation [26–28]. In addition, Sekine et al. [29] found that KLHDC10, a protein with BC box and six tandem kelch repeats, was required for H_2_O_2_-induced sustained activation of ASK1 and cell death in Neuro2A cells. Another research by Mahrour et al. [30] reported that two other kelch proteins, KLHDC2 and KLHDC3, were able to interact with Cullin2/5 proteins via their BC boxes. These information prompted us to assess whether KLHDC4 associates with cullin proteins. Immunoprecipitation assay revealed that KLHDC4 was not able to interact with Cullin2 or Cullin3 (S1A and S1B Fig).Besides, purification of KLHDC4 protein complex in human cells did not found any proteins that substantially associates with KLHDC4, none of any cullin protein family members, or other kelch proteins, have been identified in mass spectrometry data (data not shown). Structure analysis also implied that KLHDC4 lacks consensus modules mediated its interaction with cullin proteins (S1C Fig).All the evidences suggest that KLHDC4 may exert its functions other than CRL-mediated protein degradation pathway, or not even through canonical protein-protein interaction. Quantitative proteomics and DNA microarray are needed in the future to fully understand the molecular mechanism by which KLHDC4 participates in anti-apoptosis process.

## Conclusion

High level of KLHDC4 is observed in NPC patients and is associated with poorer overall survival and metastasis-free survival. Loss of KLHDC4 expression in NPC cell line leads to reduced cell proliferation, migration, invasion and tumorigenesis *in vivo*. KLHDC4 favorites tumor development partly by inhibiting cellular apoptosis.

## Supporting Information

S1 Fig KLHDC4 does not interact with Cullin2 or Cullin3. (A-B) 293T cells were transiently tranfected with plasmids encoding SFB-tagged KLHDC3, KLHDC4, KLHDC10, KEAP1, or empty vector together with plasmid encoding HA-tagged Cullin2 or Cullin3 as indicated. Cell lysates were precipitated with S-protein beads and immunoblotted with indicated antibodies. (C) Protein structures of KLHDC4, KLHDC3, KLHDC10 and KEAP1. Red rectangle: Kelch repeat; Blue hexagon: BC box; Yellow rhombus: BTB domain; Green rhombus: BACK domain.
(TIF)

## References

[1] Wei WI, Sham JS. Nasopharyngeal carcinoma. Lancet. 2005;365(9476):2041–54. 10.1016/S0140-6736(05)66698-6 . 15950718

[2] Yu MC, Yuan JM. Epidemiology of nasopharyngeal carcinoma. Semin Cancer Biol. 2002;12(6):421–9. . 12450728 10.1016/s1044579x02000858

[3] Chang ET, Adami HO. The enigmatic epidemiology of nasopharyngeal carcinoma. Cancer Epidemiol Biomarkers Prev. 2006;15(10):1765–77. 10.1158/1055-9965.EPI-06-0353 . 17035381

[4] Lo KW, To KF, Huang DP. Focus on nasopharyngeal carcinoma. Cancer cell. 2004;5(5):423–8. . 15144950 10.1016/s1535-6108(04)00119-9

[5] Ma J, Mai HQ, Hong MH, Min HQ, Mao ZD, Cui NJ, et al Results of a prospective randomized trial comparing neoadjuvant chemotherapy plus radiotherapy with radiotherapy alone in patients with locoregionally advanced nasopharyngeal carcinoma. J Clin Oncol. 2001;19(5):1350–7. . 11230478 10.1200/JCO.2001.19.5.1350

[6] Wang TJC, Riaz N, Cheng SK, Lu JJ, Lee NY. Intensity-modulated radiation therapy for nasopharyngeal carcinoma: a review. J Radiat Oncol. 2012;1(2):129–46. 10.1007/s13566-012-0020-4

[7] Fulda S, Debatin KM. Extrinsic versus intrinsic apoptosis pathways in anticancer chemotherapy. Oncogene. 2006;25(34):4798–811. 10.1038/sj.onc.1209608 . 16892092

[8] Elmore S. Apoptosis: a review of programmed cell death. Toxicol Pathol. 2007;35(4):495–516. 10.1080/01926230701320337 17562483 PMC2117903

[9] Fulda S, Debatin KM. Targeting apoptosis pathways in cancer therapy. Curr Cancer Drug Targets. 2004;4(7):569–76. . 15578914 10.2174/1568009043332763

[10] Ghobrial IM, Witzig TE, Adjei AA. Targeting apoptosis pathways in cancer therapy. CA Cancer J Clin. 2005;55(3):178–94. . 15890640 10.3322/canjclin.55.3.178

[11] Lin ML, Chen SS, Lu YC, Liang RY, Ho YT, Yang CY, et al Rhein induces apoptosis through induction of endoplasmic reticulum stress and Ca2+-dependent mitochondrial death pathway in human nasopharyngeal carcinoma cells. Anticancer research. 2007;27(5A):3313–22. . 17970076

[12] Lin ML, Lu YC, Chung JG, Li YC, Wang SG, N GS, et al Aloe-emodin induces apoptosis of human nasopharyngeal carcinoma cells via caspase-8-mediated activation of the mitochondrial death pathway. Cancer letters. 2010;291(1):46–58. 10.1016/j.canlet.2009.09.016 . 19942342

[13] Hu ZY, Zhu XF, Zhong ZD, Sun J, Wang J, Yang D, et al ApoG2, a novel inhibitor of antiapoptotic Bcl-2 family proteins, induces apoptosis and suppresses tumor growth in nasopharyngeal carcinoma xenografts. International journal of cancer Journal international du cancer. 2008;123(10):2418–29. 10.1002/ijc.23752 . 18712728

[14] Huang TS, Shu CH, Chao Y, Chen SN, Chen LL. Activation of MAD 2 checkprotein and persistence of cyclin B1/CDC 2 activity associate with paclitaxel-induced apoptosis in human nasopharyngeal carcinoma cells. Apoptosis: an international journal on programmed cell death. 2000;5(3):235–41. . 11225845 10.1023/a:1009652412399

[15] Li HL, Ye KH, Zhang HW, Luo YR, Ren XD, Xiong AH, et al Effect of heparin on apoptosis in human nasopharyngeal carcinoma CNE2 cells. Cell research. 2001;11(4):311–5. 10.1038/sj.cr.7290101 . 11787776

[16] Adams J, Kelso R, Cooley L. The kelch repeat superfamily of proteins: propellers of cell function. Trends Cell Biol. 2000;10(1):17–24. . 10603472 10.1016/s0962-8924(99)01673-6

[17] Gettemans J, Meerschaert K, Vandekerckhove J, De Corte V. A kelch beta propeller featuring as a G beta structural mimic: reinventing the wheel? Sci STKE. 2003;2003(191):PE27 10.1126/stke.2003.191.pe27 . 12865498

[18] Gupta VA, Beggs AH. Kelch proteins: emerging roles in skeletal muscle development and diseases. Skelet Muscle. 2014;4:11 10.1186/2044-5040-4-11 24959344 PMC4067060

[19] Bennett EJ, Rush J, Gygi SP, Harper JW. Dynamics of cullin-RING ubiquitin ligase network revealed by systematic quantitative proteomics. Cell. 2010;143(6):951–65. 10.1016/j.cell.2010.11.017 21145461 PMC3008586

[20] Shalem O, Sanjana NE, Hartenian E, Shi X, Scott DA, Mikkelsen TS, et al Genome-scale CRISPR-Cas9 knockout screening in human cells. Science. 2014;343(6166):84–7. 10.1126/science.1247005 24336571 PMC4089965

[21] Sizhong Z, Xiukung G, Yi Z. Cytogenetic studies on an epithelial cell line derived from poorly differentiated nasopharyngeal carcinoma. International journal of cancer Journal international du cancer. 1983;31(5):587–90. . 6852976 10.1002/ijc.2910310509

[22] Chou J, Lin YC, Kim J, You L, Xu Z, He B, et al Nasopharyngeal carcinoma—review of the molecular mechanisms of tumorigenesis. Head & neck. 2008;30(7):946–63. 10.1002/hed.20833 18446839 PMC3046044

[23] Hanahan D, Weinberg RA. Hallmarks of cancer: the next generation. Cell. 2011;144(5):646–74. 10.1016/j.cell.2011.02.013 . 21376230

[24] Huang W, Liu J, Feng X, Chen H, Zeng L, Huang G, et al DLC-1 induces mitochondrial apoptosis and epithelial mesenchymal transition arrest in nasopharyngeal carcinoma by targeting EGFR/Akt/NF-kappaB pathway. Medical oncology. 2015;32(4):115 10.1007/s12032-015-0564-4 . 25779535

[25] Sun GG, Lu YF, Fu ZZ, Cheng YJ, Hu WN. EMP1 inhibits nasopharyngeal cancer cell growth and metastasis through induction apoptosis and angiogenesis. Tumour biology: the journal of the International Society for Oncodevelopmental Biology and Medicine. 2014;35(4):3185–93. 10.1007/s13277-013-1416-5 . 24292952

[26] Kobayashi A, Kang MI, Okawa H, Ohtsuji M, Zenke Y, Chiba T, et al Oxidative stress sensor Keap1 functions as an adaptor for Cul3-based E3 ligase to regulate proteasomal degradation of Nrf2. Mol Cell Biol. 2004;24(16):7130–9. 10.1128/MCB.24.16.7130-7139.2004 15282312 PMC479737

[27] Cullinan SB, Gordan JD, Jin J, Harper JW, Diehl JA. The Keap1-BTB protein is an adaptor that bridges Nrf2 to a Cul3-based E3 ligase: oxidative stress sensing by a Cul3-Keap1 ligase. Mol Cell Biol. 2004;24(19):8477–86. 10.1128/MCB.24.19.8477-8486.2004 15367669 PMC516753

[28] Gorokhova S, Bibert S, Geering K, Heintz N. A novel family of transmembrane proteins interacting with beta subunits of the Na,K-ATPase. Hum Mol Genet. 2007;16(20):2394–410. Epub 2007/07/04. 10.1093/hmg/ddm167 . 17606467

[29] Sekine Y, Hatanaka R, Watanabe T, Sono N, Iemura S, Natsume T, et al The Kelch repeat protein KLHDC10 regulates oxidative stress-induced ASK1 activation by suppressing PP5. Molecular cell. 2012;48(5):692–704. 10.1016/j.molcel.2012.09.018 . 23102700

[30] Mahrour N, Redwine WB, Florens L, Swanson SK, Martin-Brown S, Bradford WD, et al Characterization of Cullin-box sequences that direct recruitment of Cul2-Rbx1 and Cul5-Rbx2 modules to Elongin BC-based ubiquitin ligases. J Biol Chem. 2008;283(12):8005–13. 10.1074/jbc.M706987200 . 18187417

