# Supplementary figures and images for: Upregulation of KLHDC4 Predicts a Poor Prognosis in Human Nasopharyngeal Carcinoma
Source: PLoS One. 2016 Mar 31;11(3):e0152820. doi: 10.1371/journal.pone.0152820 (PMC4816273; doi:10.1371/journal.pone.0152820)

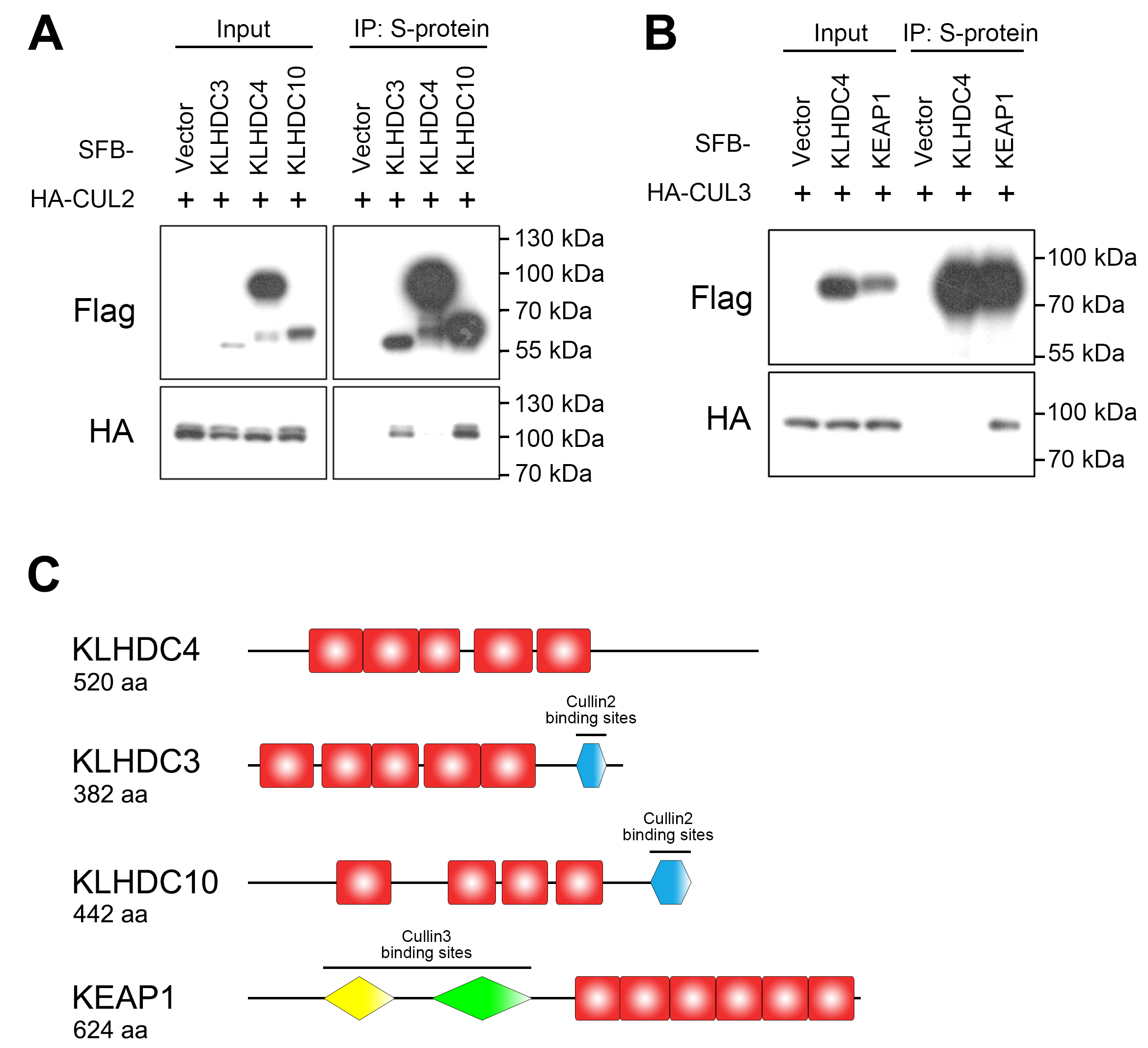

Supplement: S1 Fig — (A-B) 293T cells were transiently tranfected with plasmids encoding SFB-tagged KLHDC3, KLHDC4, KLHDC10, KEAP1, or empty vector together with plasmid encoding HA-tagged Cullin2 or Cullin3 as indicated. Cell lysates were precipitated with S-protein beads and immunoblotted with indicated antibodies. (C) Protein structures of KLHDC4, KLHDC3, KLHDC10 and KEAP1. Red rectangle: Kelch repeat; Blue hexagon: BC box; Yellow rhombus: BTB domain; Green rhombus: BACK domain. (TIF) [file pone.0152820.s001.tif]
